# Increased risk of death following recurrent ketoacidosis admissions: a Brazilian cohort study of young adults with type 1 diabetes

**DOI:** 10.1186/s13098-023-01054-5

**Published:** 2023-04-28

**Authors:** Sarah S. Santos, Luana A. L. Ramaldes, Patricia M. Dualib, Monica A. L. Gabbay, João R. Sá, Sergio A. Dib

**Affiliations:** Department of Medicine, Endocrinology Division, Diabetes & Endocrinology Center, Escola Paulista de Medicina, Universidade Federal de Sao Paulo, Caixa Postal 20266/CEP: 04022-001, Sao Paulo, Brazil

**Keywords:** Diabetic ketoacidosis, Type 1 diabetes mellitus, Recurrent diabetic ketoacidosis, Mortality

## Abstract

**Background:**

Recurrent DKA (rDKA) remains an acute type 1 diabetes complication even in post-insulin era. This study aimed to analyze the predictors and effects of rDKA on the mortality of patients with type 1 diabetes.

**Methods:**

Patients hospitalized (n = 231) wih diabetic ketoacidosis (between 2007 and 2018) were included. Laboratorial and clinical variables were collected. Mortality curves were compared in four groups: diabetic ketoacidosis as a new-onset type 1 diabetes (group A), single diabetic ketoacidosis episode after diagnosis of type 1 diabetes (group B), 2–5 diabetic ketoacidosis events (group C), and > 5 diabetic ketoacidosis events during follow-up period (group D).

**Results:**

During the follow-up period (approximately 1823 days), the mortality rate was 16.02% (37/231). The median age at death was 38.7 years. In the survival curve analysis, at 1926 days (5 years), the probabilities of death were indicated by ratios of 7.78%, 4.58%, 24.40%, and 26.63% in groups A, B, C, and D, respectively. One diabetic ketoacidosis episode compared with ≥ 2 events had a relative risk of 4.49 (p = 0.004) of death and > 5 events had 5.81 (p = 0.04). Neuropathy (RR 10.04; p < 0.001), retinopathy (relative risk 7.94; p < 0.01), nephropathy (RR 7.10; p < 0.001), mood disorders (RR 3.57; p = 0.002), antidepressant use (RR 3.09; p = 0.004), and statin use (RR 2.81; p = 0.0024) increased the risk of death.

**Conclusions:**

Patients with type 1 diabetes with > 2 diabetic ketoacidosis episodes have four times greater risk of death in 5 years. Microangiopathies, mood disorders, and use of antidepressants and statins were important risk factors for short-term mortality.

## Background

Diabetic ketoacidosis (DKA) is still the leading cause of death and morbidity and imposes major demands on the health of patients with type 1 diabetes (T1D). At initial diagnosis, the incidence of DKA ranges from 13 to 80% [1–4] and varies according socioeconomic country backgrund. In Brazil, a multicenter study demonstrated that 42.3% of T1D individuals presented DKA at diagnosis [4] noncompliance is the major precipitating factor of DKA [5, 6], which is an important cause of death [7].

Questionably, even with the low mortality rate (1–2%) in acute events [1, 8, 9], mainly justified by guidelines and institutional protocols to diagnose and treat this condition and avoid unfavorable outcomes, evidence reveals that recurrent DKA (rDKA) throughout the life of a patient with T1D can be an important predictor of mortality [10, 11]. In patients with chronic T1D, chronic hyperglycemia, continuous subcutaneous insulin infusion dysfunction, smoking, alcohol consumption, and diabetic nephropathy were associated with recurrent rDKA [12]. This condition can be also a part of “brittle diabetes” [10, 13–18]. Nevertheless, rDKA, which is not yet precisely defined, can account for up to > 50% of DKA cases in huge hospitals [19]. rDKA could be associated with substantial mortality rates during follow-up after hospital discharge [11, 16] and partially with an increased risk of suicide attempts [20, 21].

In patients with rDKA, death usually occurs relatively early (4th decade of life), following hospital discharge, in those with > 5 DKA admissions [11]. Recent data from a US cohort showed that 21.6% of DKA cases were recurrent, and the odds of death increased with the number of hospital admissions for DKA [16]. These data are in contrast with those recorded in 1991–2010; with improved access to care for patients with diabetes, including the free provision of insulin [22]. Thus, to prevent future admissions and reduce the mortality rate, determining the characteristics of patients at high risk for rDKA is essential to the implementation of an adequate follow-up program.

To the best of our knowledge, no study has addressed this issue in the Brazilian context, which is relevant because the risk factors of rDKA may have particularities in a patient population who have universal access to a public healthcare system. Our teaching hospital in Sao Paulo City (SP, Brazil) serves a diverse and underserved inner-city population with high rates of rDKA. Therefore, this study aimed to retrospectively analyze patient factors associated with rDKA admissions and assess the risk of mortality in the years following a short-time hospital discharge.

## Methods

### Data source

We conducted a retrospective cohort study of patients with T1D who had a DKA episode admitted between 2007 and 2018 in a Southeast Brazilian Public University teaching hospital and registered in the electronic medical record database.

### DKA definition

In this study, a DKA episode in T1D was defined as having inpatient hospital encounters with International Classification of Diseases 10th revision (ICD-10) codes of E10.1 and E14.1, which included ketoacidosis with the fourth character code 1 (diabetic acidosis or diabetic ketoacidosis).

**Fig. 1:**
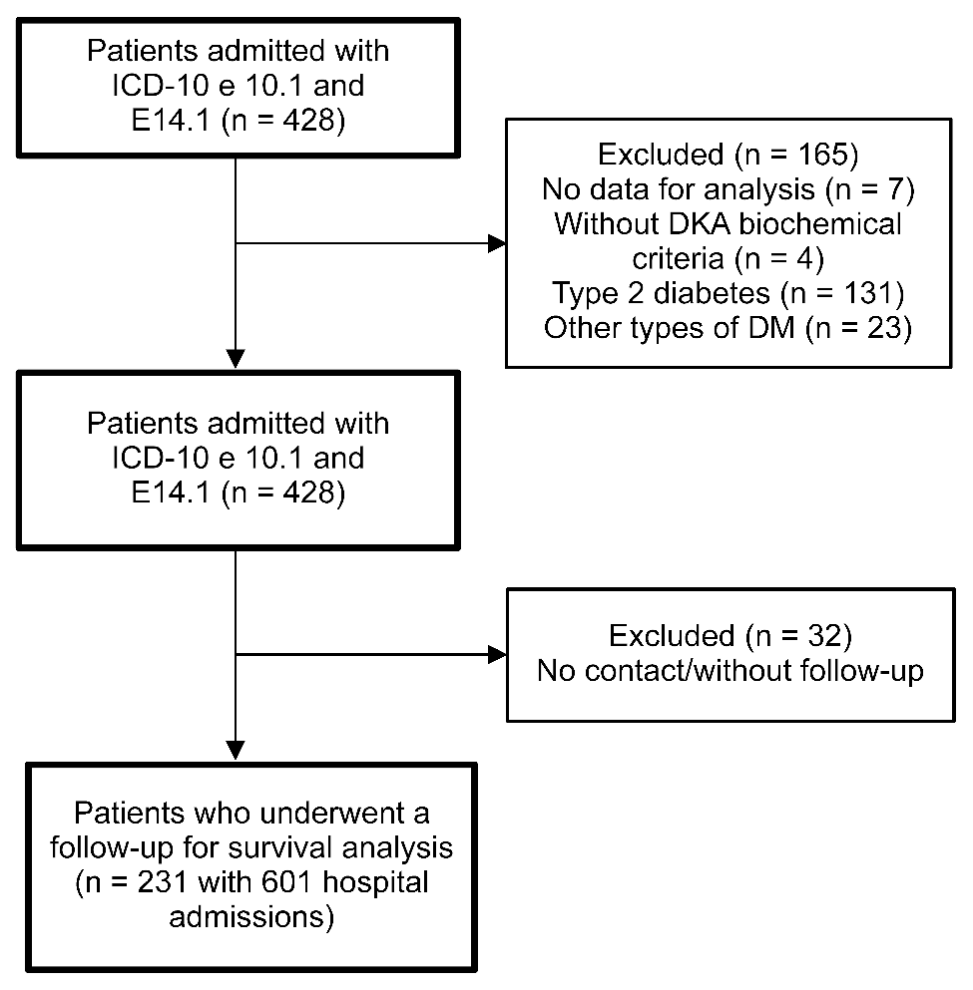
Patients selected for the study

Considering that ICD-10 coding could not exactly classify the diabetes type, each medical record was revisited to ensure better selection of only patients with T1D. From a total of 428 patients identified during the study period, we excluded those who during follow-up were diagnosed with other types of diabetes or despite the code did not fulfill the ketoacidosis criteria (n = 165) and who could not be available for follow-up (n = 32). If there was more than one episode of ketoacidosis in the same individual, the variables from the first episode were considered for the statistical analysis (Fig. 1).

Clinical and laboratory data from 231 patients were collected from hospital records admissions data. These data included age at admission, sex, glycemia, serum pH, bicarbonate, sodium, potassium, leukocyte count, current antidiabetic treatment, age at diabetes diagnosis, presence of diabetes chronic complications (retinopathy, nephropathy, and neuropathy according to the protocol published elsewhere [23]), statin use, hemoglobin A1C (HbA1c) and serum C-peptide closest to the DKA event, length of hospital stay, total number of hospitalizations in the studied period, use of illicit drugs, smoking, alcoholism, and antidepressant use.

### Mortality risk

To evaluate the mortality after hospital discharge, patients were followed up through appointments, telephone call, or virtual contacts to check for the patient’s status. They were invited to participate in this study, and written informed consent was obtained. The study was approved by the institutional review board (no. 96720818.6.0000.55.05).

The follow-up period began on the day of DKA admission and ended with the patient’s death or on the contact date. From the 231 participants selected (601 hospital admissions for DKA), a survey analysis was conducted to compare groups with event recurrence, accounting for a median 5-year follow-up time of 1823 days. To compare mortality curves, the patients were divided into four groups according to the number of DKAs during the study period: DKA as a new-onset T1D (group A), only a single DKA episode after T1D diagnosis (group B), 2–5 DKA episodes (group C), and > 5 DKA episodes (group D) during the follow-up period. We also analyzed the clinical and laboratory characteristics of these four groups and compared the risk between them.

### Statistical analysis

In the descriptive analysis, continuous variables were expressed as summary measures, whereas categorical variables were expressed as percentages. For the comparison of two groups of continuous variables, the t-test was used for variables that followed a normal distribution (Anderson–Darling test), whereas for those without a normal distribution, nonparametric Mann–Whitney and Brunner–Munzel tests were used for homogeneous and heterogeneous variables, respectively (Bartlett test). For categorical variables, Fisher’s exact test was used. For the comparison of several groups of continuous variables, the analysis of variance was used for variables that followed a normal distribution (Anderson–Darling test), whereas for those without a normal distribution, Kruskal–Wallis and nonparametric Levine tests were used for homogeneous and heterogeneous variables, respectively (Bartlett test). For multiple comparisons (2 × 2), parametric and nonparametric Tukey tests were performed for variables that follow and do not follow a normal distribution, respectively. The Kaplan–Meier method was used to make survival graphs, and the log-rank, Gehan–Breslow and Tarone–Ware methods were used to analyze the difference between the survival curves, with an estimated progression of up to 5000 days. Cox regression was used to perform the survival analysis. The level of significance was 0.05. One-tailed (right and left) and two-tailed hypotheses were considered. Software R version 3.6.0 was used to perform all analyses.

## Results

A total of 231 patients with T1D were followed over a median of 1823 days (almost 5 years) after their DKA admission. Among the patients, 50.6% were women. The age at DKA episode was 26 (interquartile range [IQR] 17–37.5) years. The HbA1c was 11.5% [IQR 9.70–13.45]. Moreover, 44.5% of the patients had retinopathy, 33.7% had neuropathy, 32.5% had nephropathy, 16.7% were statin users, 53.2% had mood disorders, 18.6% were smokers, 24.7% were alcoholic, and 20.6% were illicit drug users.

In this study, 51% of the population had one DKA episode, 17.7% (41/231) were newly diagnosed with T1D, 33.0% (76/231) had only one DKA episode after T1D diagnosis, 36.3% (84/231) had 2–5 DKA episodes, and 13.0% (30/231) had > 5 episodes.

### Treatment before the DKA episode

Regarding the diabetes treatment that had been used before hospital admission, 21.0% did not use any treatment for DM (those with new-onset T1D and those who stopped insulin treatment completely), 12.6% used only basal insulin, 47.8% used human insulin in basal/bolus regimen, 13.0% received basal/bolus regimen with insulin analogs, 3.9% used only oral medications, and 1.7% were using an insulin pump.

### Mortality after recurrent diabetes ketoacidosis

In this study, 37 patients (16.02%) died during the study period. Of these 37 deaths, 81.0% (30/37) occurred in patients with > 2 DKA episodes; specifically, 8.1% (3/37) in group A, 10.8% (4/37) in group B, 59.4% (22/37) in group C, and 21.6% (8/37) in group D. Considering all deaths, the median age at death was 38.7 (IQR 28.43–48.91) years, and the diabetes duration was 19.2 (IQR13.67–23.80) years.

### Variables associated with death during follow-up

The number of DKA hospitalizations and other variables such as age at DKA presentation (years), diabetes duration (years), C-peptide levels, retinopathy, mood disorders, neuropathy, nephropathy, statin use, and antidepressant use were different between the survivors and nonsurvivors (Table 1).

**Table 1:** Variables of survivors vs. nonsurvivors among patients with T1D and rDKA

Variables	Survivors (n = 194)	Nonsurvivors (n = 37)	p value
Number of DKA hospitalizations	1 (1–2)	3 (2–4)	< 0.001 ^1^
Age at DKA presentation (years)	24 (16–34)	36 (25–47)	< 0.001 ^1^
Diabetes duration (years)	4 (0–10)	15 (6–20.2)	< 0.001 ^2^
Bicarbonate level (mEq/L)	7.3 (3.6–11)	10.4 (7.3–12.1)	0.003 ^1^
Serum urea (mg/dL)	39 (26–55)	81.5 (50.25 − 105.5)	< 0.001 ^2^
HbA1c (%)	11.9 (10.12–13.47)	10.3 (8.7–12.5)	0.138 ^3^
HbA1c (mmol-mol)	107 (87–123)	89 (72–113)	**
C-peptide (ng/mL)	0.2 (0.01–0.62)	0.01 (0.01–0.05)	0.004 ^2^
Retinopathy	58 (35.2%)	32 (86.5%)	< 0.001^4^
Mood disorders	82 (47.7%)	28 (80.0%)	0.001^4^
Neuropathy	36 (22.5%)	30 (83.3%)	< 0.001^4^
Nephropathy	35 (21.9%)	29 (78.4%)	< 0.001^4^
Statin use	20 (12.1%)	14 (37.8%)	< 0.001^4^
Antidepressant use	37 (22.2%)	20 (54.1%)	< 0.001^4^

^1^ Mann–Whitney test, ^2^ Brunner–Munzel, ^3^ t-test, ^4^ Fisher test; Median (IQR), and N (%) ** not tested

### Survival Kaplan–Meier curve of the number of DKA episodes and mortality

**Fig. 2:**
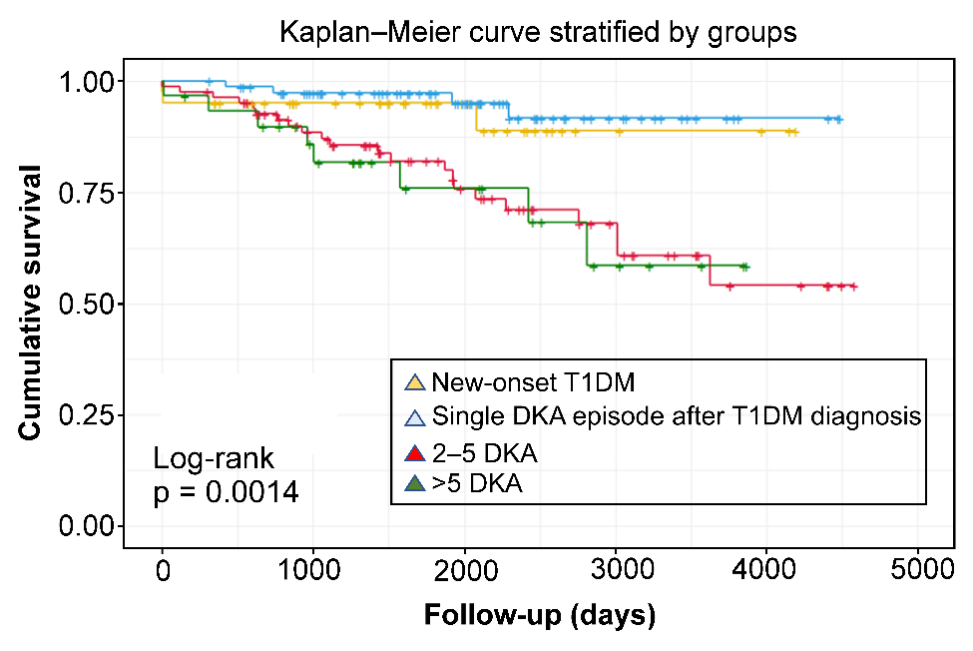
Survival curve according to the number of DKA in patients with T1D

When plotted on the survival curve with the Kaplan–Meier method, a difference was found between the curves of the group with one episode (both new-onset T1D and single DKA episode for other causes) and the group with > 2 episodes and also in the plotted curves comparing groups A, B, C and D. Group C almost had the same mortality tendency as group D; thus, from two episodes of DKA, the mortality risk is higher (Fig. 2).

Survival curves were evaluated with the Cox regression method, and at 1926 days (about 5 years), groups A, B, C, and D had probabilities of death indicated by hazard ratios (HR) of 7.78%, 4.58%, 24.40%, and 26.63%, respectively. When projected further in the long term, in approximately 4584 days (12 years), the risk increases consistently over time, with HR of 17.98%, 10.59%, 56.35%, and 61.48%, respectively).

### Regression analysis of variables and death at follow-up

In the regression analysis through the simple Cox regression method, we found that were an increased risk of death in T1D with one DKA episode (including group A and B) vs. C and D, ≥ 2 episodes RR 4.49 [IQR 1.75–19.30] p = 0.004.We also compared C and D groups comparing with a single DKA. Microangiopathy diabetic chronic complication (neuropathy, retinopathy, and nephropathy), mood disorders, antidepressant use, statin use, and age at DKA diagnosis were associated with death. These data are shown in Table 2.

**Table 2:** RRs of patients with T1D for death following DKA for all independent predictive variables from the Cox regression analysis

Variables	RR	95% CI for HR		*p* value
		Lower	Upper	
2–5 DKA admissions^a^	5.32	1.83	15.46	0.002
> 5 DKA admissions^a^	5.81	1.75	19.30	0.004
≥ 2 DKA admissions^b^	4.49	1.97	10.23	0.004
Age at DKA presentation	1.04	1.02	1.06	< 0.001
Neuropathy	10.04	4.18	24.15	< 0.001
Retinopathy	7.94	3.09	20.40	< 0.001
Nephropathy	7.10	3.24	15.56	< 0.001
Mood disorders	3.57	1.56	8.18	0.002
Antidepressant use	3.09	1.62	5.91	< 0.001
Statin use	2.81	1.44	5.48	0.002

^a^ Compared with a single DKA, ^b^ compared with one episode DKA (new-onset T1D or only a single DKA episode)

## Discussion

In this retrospective study of 231 young adults with T1D who experienced DKA, recurrence of ≥ 2 episodes was related to a substantial (four times higher than the background population) short-term (next 5 years) risk of death. The major risk factors contributing to death were the presence of diabetic microangiopathy (neuropathy, retinopathy, or nephropathy), mood disorders, antidepressant use, statin use, and age at DKA diagnosis.

According to other data including our country, 17% of DKA cases were due to new-onset T1D [24–26]. Despite the total mortality of 16.02%, we found a low rate of inpatient mortality from DKA (2.16%), which is almost satisfactory according to most recent studies [19].

When subcategorized by the number of hospitalizations, the highest probability of death was found in the group with 2–5 episodes of DKA within 5 years of follow-up. This group also had the highest mortality rate (60%).

Most patients were young adults at the time of DKA episodes. Thus, in those with > 5 hospitalizations, the median age was 19 years, suggesting that this age group is critical for rDKA. A young age at T1D diagnosis and psychological changes during adolescence are high-risk factors for rDKA if patients are not well supervised during this period [6–26].

In our population, although the study was conducted in a developing country, our gross mortality data were similar to those in studies conducted in developed countries, further reinforcing the global difficulty of diabetes control in these individuals and the concept that they may represent a different T1D group with specific demands for care. That is, in our study, the mortality curve projection indicated that those who have > 2 DKA episodes during their lifetime have a greater mortality risk, specifically those with rDKA. Thus, this patient group must receive personalized follow-up and different from other patients with T1D, considering that mortality and its psychosocial aspects varied and require different monitoring [13, 27].

In this study, the median age of mortality was < 40 years old. If compared with the average Brazilian life expectancy, these patients lost on average three decades of life [28].

The main triggers of DKA noted in this study were the lack of adherence to treatment (47%). Theoretically, considering that a large proportion of the studied patients were on standard treatment with multiple doses of human insulin or analogs (approximately 60%) and just a few had a treatment considered inappropriate for T1D (15.5% were using oral medications or only basal insulin), insulin compliance is probably the main goal to prevent rDKA. More importantly, in our National Health System, patients have free access to insulin. Another factor that is related to treatment compliance is the significant difference between psychiatric disturbances and antidepressant use in nonsurvivors when compared with survivors. According to our data, antidepressant users are approximately three times more likely to die than those who do not. In UK [29] and US [17] series, higher DKA rates were found in patients who have depression, received antipsychotic medications, and had substance abuse.

On a pathogenetic point of view regarding T1D, nonsurvivors had lower levels of C-peptide, corroborating both a longer time of T1D diagnosis and a lower insulin reserve in this group. This finding is in line with those in another study [30], showing that worse beta cell function is associated with rDKA.

In our assessment of risk factors, we found that neuropathy, nephropathy, and retinopathy increase 7–10 times the mortality risk of individuals with rDKA. Despite being an expected finding, the presence of chronic complications increases the risk of death [24].

As statin use was a risk factor for death (RR 2.8), the manifested and residual cardiovascular disease is a potential determinant of worse outcomes. Patients diagnosed with T1D before the age of 10 years may have 30 times increased risk of coronary heart disease and acute myocardial infarction in the early adult years [31].

The strengths of the study were related to the single-center setting and double-checking of medical records to exclude individuals who, despite having ICD code for ketoacidosis, do not have T1D, considering our institutional clinical criteria for the classification of T1D.

The vast majority of studies on rDKA do not take into account that research based solely on ICD-10 may result in discrepancies and errors related to diabetes classification, biochemical criteria of DKA, coding accuracy [32], and changes in ICD classification over the years [33]. The use of entry codes alone, as a big data analysis, may result in having a larger sample of patients [25], but may not include patients with T1D alone. Thus, we try to minimize the inclusion of patients who were classified inadequately as having T1D or who did not have the biochemical criteria for DKA.

Moreover, as this study relied on data from electronic medical records, with data sources collected primarily in hospitals, this study may have some biases regarding the validity of information despite double-checking.

The survival analysis was based on previous studies and a pilot analysis. Some patients were lost and were removed from the analysis (n = 32). However, these were analyzed statistically, and no variable with a statistical difference was found in the total analysis group (n = 231).

As the characteristics of our patients were broadly comparable with those in several other studies [34, 35], we can say that our findings have wider applicability regarding the significant associationss in the risks of both rDKA and subsequent mortality, despite the relatively small sample size.

## Conclusions

The results of this study revealed that > 2 DKA episodes in a patient with T1D is an important risk factor for short-term mortality, and the major risk factors were the presence of diabetic microangiopathy, mood disorders, age at DKA diagnosis, and diabetes duration.

In understanding the social and psychological scenario that this patient group, individualization and early identification of rDKA are necessary to establish a personalized follow-up with a multidisciplinary approach [36] and avoid unfavorable outcomes, excessive healthcare costs, and short lifetime in individuals with T1D.

## Data Availability

The datasets generated and was analysed during the current study are available in the UNIFESP repository.

## List of abbreviations

DKA: diabetic ketoacidosis
HR: hazard ratio
IQR: interquartile range
rDKA: recurrent DKA
T1D: type 1 diabetes
HbA1c: hemoglobin A1C
